# Supplementary material for: The insulin sensitivity Mcauley index (MCAi) is associated with 40-year cancer mortality in a cohort of men and women free of diabetes at baseline
Source: PLoS One. 2022 Aug 3;17(8):e0272437. doi: 10.1371/journal.pone.0272437 (PMC9348742; doi:10.1371/journal.pone.0272437)
Supplement: S1 Table — (PDF) [file pone.0272437.s001.pdf]

Table S-1: Distribution of malignancy attributed causes of death

**Type of cancer**	**Count, n (%)**
All digestive	94 (35.7)
Colorectal	33 (12.5)
All Genito - Urinary	44 (16.7)
Prostate	18 (6.8)
Lung	31 (11.7)
Hematopoietic and lymphoid	26 (9.8)
Breast	21 (7.9)
Other	47 (17.8)
Total	263
